# A novel technique for low-rectal cancer: Pushing the anus in laparoscopic radical resection

**DOI:** 10.1097/MD.0000000000043267

**Published:** 2025-07-04

**Authors:** Rui Yang, Yuyi Yang, Yaxing Deng, Qingqiang Yang

**Affiliations:** a Department of General Surgery (Gastrointestinal Surgery), The Affiliated Hospital of Southwest Medical University, Luzhou, Sichuan Province, China; b The First School of Clinical Medicine, Southern Medical University, Guangzhou, Guangdong Province, China.

**Keywords:** laparoscopic total mesorectal excision, low-rectal cancer, pushing the anus, sphincter-preserving surgery

## Abstract

In sphincter-preserving surgery for low-rectal cancer, it is significant to reduce the number of stapler cartridges and the incidence of anastomotic leakage. Therefore, this study introduces a new and safer technique in laparoscopic radical resection of low-rectal cancer to reduce the number of stapler cartridges used during operation and reduce the incidence of postoperative anastomotic leakage. Information was collected on 237 patients with rectal cancer from the Affiliated Hospital of Southwest Medical University between January 2015 and July 2020. In 151 cases, the surgeon used the stapler cartridges (Ethicon Intraluminal Linear Staplers EC60A, Ethicon) to transect the edge of tumor of the rectum (conventional surgery group; n = 151). In other cases, besides applying the stapler cartridges, the surgeon had the assistant push the anus forward from the perineum during the process of transecting the rectum (pushing the anus group; n = 62). The postoperative outcomes and complications were compared between the 2 groups. As a result, in terms of the number of the stapler cartridges, the pushing the anus group was less than the conventional surgery group (*P* < .001). Moreover, the incidence of anastomotic leakage in the pushing the anus group is lower than that in the conventional surgery group (*P* = .043). These results proved that with pushing the anus forward during the process of transecting the rectum, the sphincter-preserving surgery can be performed more safely.

## 1. Introduction

Rectal cancer is the eighth most common malignancy in the world and poses a great threat to human health.^[1]^ Among them, low-rectal cancer accounts for 75% of all rectal cancers and constitutes a severe global public health burden.^[2]^ Low-rectal cancer is generally considered as ≤5 cm from the lower edge of the tumor to the anal verge.^[3]^ At present, surgery is still the main means of treating low-rectal cancer. Laparoscopic technology has made great progress in recent years, such as the application of the circular stapler and the double stapler techniques, and more anus is preserved in an increasing number of patients with low-rectal cancer. However, the sphincter-preserving surgery is more challenging than abdominoperineal resection, and the risk of postoperative anastomotic leakage remains a problem for surgeons.^[4]^ Due to the narrow space around the rectum, it brings difficulty to the movement of the rigid instruments of laparoscopy and the surgical field of view, which makes a lack of standardization of the approach and procedure of laparoscopic radical resection of low-rectal cancer.^[2]^ Although there are now many guidelines or expert consensus on rectal cancer surgery, such as total mesenteric resection (TME), root lymph node dissection of the inferior mesenteric artery (IMA), pelvic autonomic nerve preservation, etc, laparoscopic radical resection of low-rectal cancer is still highly dependent on the experience of the surgeon.^[5]^

In traditional surgery, due to the narrow and deep anatomy of the pelvic cavity, surgeons often need to use additional stapler cartridges when disconnecting the rectum at the lower edge of the tumor. Besides, increased tension caused by excessive straining of the rectum has a negative influence on the nail formation of linear cutting closure and may lead to tearing of the seromuscular layer, as well as affect the blood supply of the broken end of the rectum. All of these factors increase the risk of anastomotic leakage. Therefore, how to perform surgery more safely and conveniently and reduce anastomotic leakage is an important issue for surgeons. There are various methods to prevent anastomotic leakage after low anterior resection in patients with rectal cancer, such as defunctioning loop ileostomy, 3-row circular staplers, low IMA ligation, and the transanal drainage tube use.^[6,7]^ But these methods also have limitations, such as loop ileostomy requiring secondary surgery and perianal pain in patients with transanal drainage tube. Therefore, based on the surgical experience of this research team, this paper introduces a new technique in laparoscopic resection of low-rectal cancer. During rectal transection, the assistant pushes the anus forward from the perineum toward the patient’s head to bring the surgical plane closer to the entrance to the pelvis for a wider surgical space. To reduce the incidence of anastomotic leakage during sphincter-preserving surgery with fewer stapler cartridges. The details and evaluation of this technique are described below.

## 2. Patients and methods

### 2.1. Patients

Information was collected on 237 patients with rectal cancer from the Affiliated Hospital of Southwest Medical University between January 2015 and July 2020. Inclusion criteria: preoperative diagnosis of rectal adenocarcinoma by electronic colonoscopy and pathological biopsy; nuclear magnetic resonance image suggested that the lower margin of the tumor was 2.5 to 5 cm from the anal margin; laparoscopic radical resection of rectal cancer performed by the same surgical team; 18 years ≤ age ≥80 years; primary rectal cancer confirmed by imaging examination and no distant metastasis. After excluding patients with incomplete clinical data, 237 study subjects were included. Of these, a protective ileostomy was performed in 18 patients due to minor colonic edema, and in 6 patients, the splenic flexure was released to obtain a bowel tube of sufficient length for anastomosis because the marginal vessels were found to be incompetent intraoperatively. To avoid the above factors influencing the surgical outcome, these 24 patients were excluded and 213 patients were finally included in the study. All patients underwent active peri-operative preparation including blood glucose control, correction of hypoproteinemia, routine smoking cessation and so on. In addition, all patients had adequate preoperative bowel preparation, and no patient required emergency surgery for acute intestinal obstruction. The study divided all subjects into 2 groups based on whether the assistant pushed the anus during transecting the rectum, including the pushing the anus group (n = 62) and the conventional surgery (n = 151). This study was approved by the Ethics Committee of the Affiliated Hospital of Southwest Medical University and was in accordance with the Declaration of Helsinki.

### 2.2. Clinical data

We collected and sorted out the patient’s clinical data, including age, gender, body mass index, American Society of Anesthesiology (ASA) classification, PT/N stage, tumor size, neoadjuvant therapy and distance from the lower edge of the tumor to the anal verge. Intraoperative conditions and indicators related to postoperative recovery of patients. Including intraoperative bleeding, first postoperative meal, first anal exhaust, postoperative hospital stay, operative time, number of stapler cartridges, the average distance of anus moved forward (the pushing the anus group). Moreover, we also studied the occurrence of postoperative complications. Including anastomotic leakage, anastomotic bleeding, anastomotic stenosis, pulmonary complication, postoperative obstruction, urinary retention, chylous ascites, and abdominal infection. The anastomosis leakage is diagnosed and graded according to the proposal by International Study Group of Rectal Cancer (ISREC).^[8]^

### 2.3. Surgical techniques

**Figure 1.**
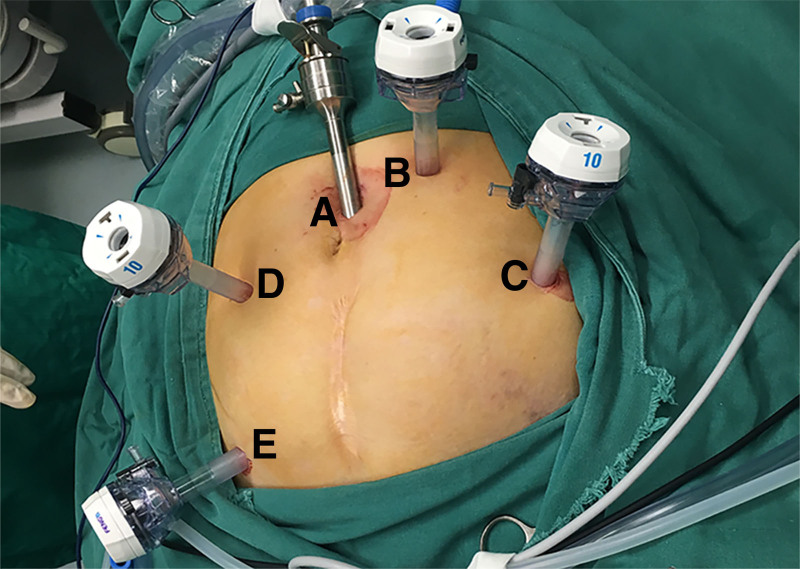
Placement of trocar. (A) 10-mm metal trocar for the scope. (B) 10-mm trocar. (C) 10-mm trocar. (D) 10-mm trocar. (E) 12-mm trocar.

Under the condition of general anesthesia, the patient was placed in the Lloyd-Davies position. The master knife doctor stood on the right side of the patient, while the first assistant stood on the left side of the patient and the laparoscope operator stood on the patient’s head side. After pneumoperitoneum was established, a 10 mm camera trocar was introduced blow the umbilicus. Four additional trocars were created in the lower abdomen (Fig. 1).

**Figure 2.**
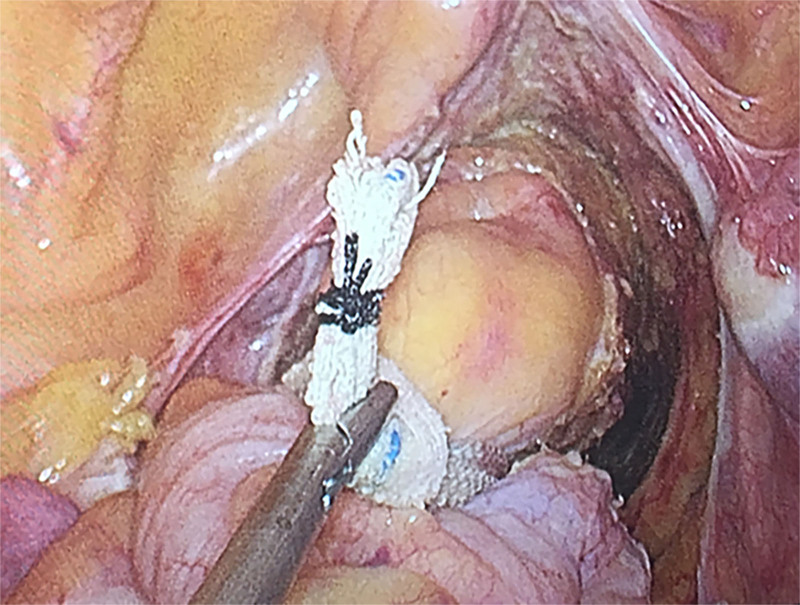
Pulling the rectum with gauze. Wrap the gauze around the rectum, tighten it and pull it upward with the needle holder to maintain enough tension to facilitate surgical operation.

In the Trendrenberg position, the small intestine is displaced from the pelvis by leaning the body to the right, concentrating as much as possible up the abdomen to facilitate surgical manipulation. The sigmoid colon was detached from the abdominal wall. And then the foot of the sigmoid mesocolon was dissociated from the medial to the lateral. Lymph nodes in the root of the IMA were cleared and the left colonic artery was preserved. Then, the IMA and the inferior mesenteric vein were ligated and transected. The lateral colonic attachments were then loosened along the white line of Toldt to completely mobilize the lower descending sigmoid colon. And take care to protect the ureter, the inferior ventral nerve and the pelvic parasympathetic plexus. Like using a plastic beaded form urinary Foley catheter bag hanger, we used gauze to pull the rectum to maintain its tension and make it easier to operate (Fig. 2).^[9]^

**Figure 3.**
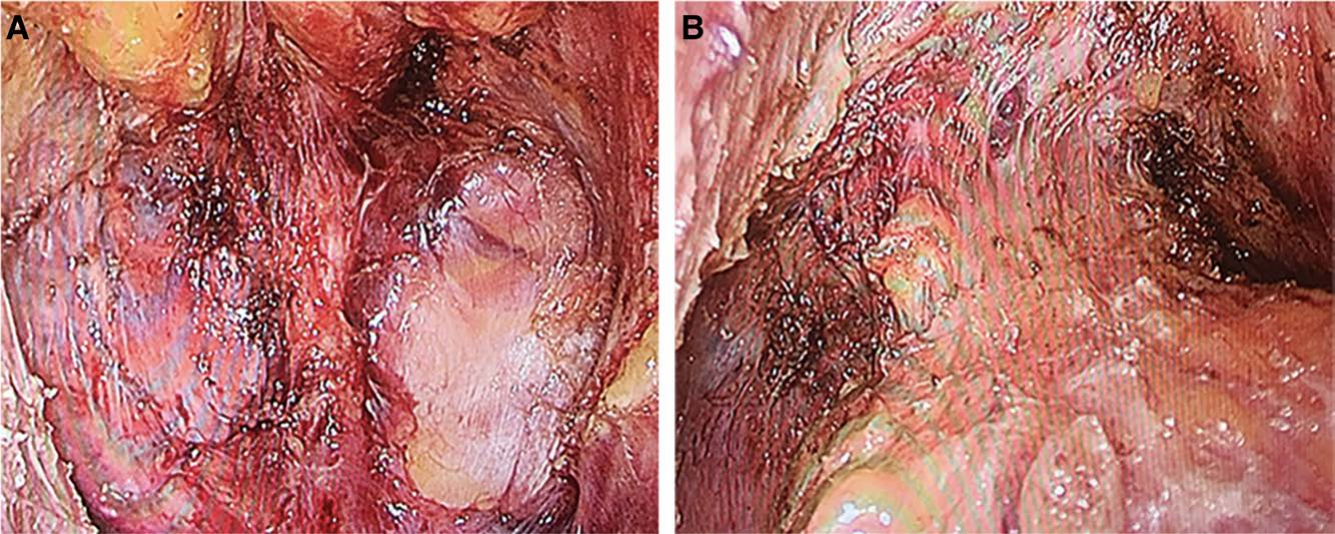
Pelvic floor condition after dissociation. (A) Posterior rectum. (B) Anterior rectum.

Following the principles of TME as described by Heald et al, the rectum was freed to the lower edge of the tumor.^[10]^ Additionally, performing partial or full intersphincteric resection (ISR) depending on the intraoperative situation (Fig. 3).^[11]^ ISR consisted of 2 phases: abdominal and perineal. The transabdominal part began with an artery first approach (IMA), followed by medialto-lateral mobilization of the descending colon, division of the inferior mesenteric vein, and TME with autonomic nerve preservation. Subsequently, intersphincteric dissection was performed in the transanal phase in patients in ISR. For transanal approach, locate a self-retaining retractor at the anus or fix and pull the anus through the suture.^[12]^ After routine disinfection, a circular incision of the anal canal was performed 1 cm below the tumor, removing one-third or half of the internal anal sphincter. The the intersphincteric plane (ISP) was then dissected circumferentially.^[13]^ Next, an anastomosis was performed on the distal anus and the proximal rectum. The final tumor specimen was removed through the anus.

**Figure 4.**
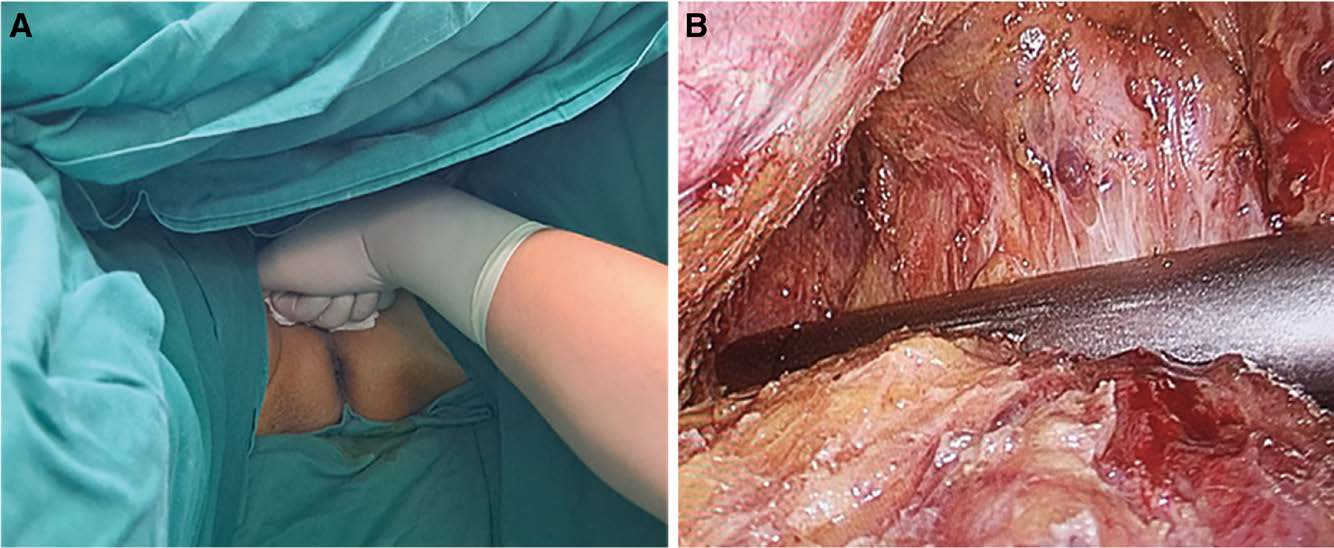
Transecting the rectum while pushing the anus. (A) Pushing the anus by assistant (position of pushing the anus: in male patients, it is between the upper edge of the anus and the lower edge of the scrotum; in female patients, it is between the upper edge of the anus and the lower edge of the labia majora). (B) Transection of the rectum below the tumor.

**Figure 5.**
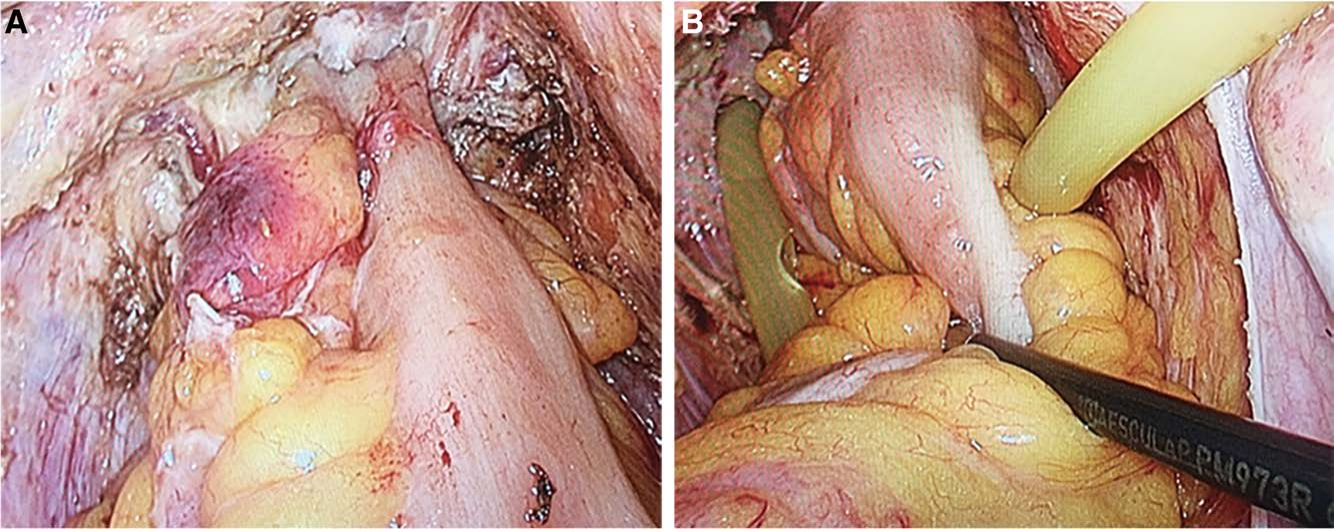
The completion of anastomosis. (A) Digestive tract reconstruction. (B) Placement of plasma drainage tube.

The next step is to perform the disconnection of the rectum, namely cutting the rectum at the lower edge of the tumor. In the traditional laparoscopic radical resection of low-rectal cancer, the surgeon directly uses a linear cutting closure through the rectum and separate the rectum. However, the low location of the tumor and its distance from the entrance to the pelvis make the surgical space narrow, which is more prominent in male patients with a narrow pelvis and in obese patients. The narrow surgical space has a great impact on the field of view and the operation of the surgeon, which is one of the important reasons for the complications of anastomotic leakage and anastomotic bleeding after surgery. On the basis of traditional surgery, the new technology introduced in this study allows the assistant to place a folded gauze on the patient’s perineum, and then the fist applies upward pressure on the gauze to make the perineum move forward to the patient’s head, and the core action point is located 1 to 2 cm above the anus (Fig. 4). In this way, during the operation, the plane of the rectum that is about to be severed can be moved upward, bringing it closer to the entrance of the pelvis, thus obtaining a wider surgical field of view and a larger operating space. At the same time, the first assistant can pull the gauze wrapped in the rectum upward, so as to ensure that the intestine has the appropriate tension for disconnection. Then, a surgical incision of about 5 to 8 cm is made in the left lower abdomen, and the proximal tumor and intestines are placed outside the abdominal cavity through this incision to facilitate the removal of the tumor. This process requires attention to protect the marginal vessels to ensure adequate blood supply to the anastomosis. In addition, for the patients whose resection margins of the distal end of the tumor were <2 cm, the cutoff end of specimens should be sent for intraoperative frozen biopsies to make sure the margin is negative. Then, the tumor was completely excised and an anastomotic anvil was placed in the proximal bowel tube. Subsequently, the incision was closed and the pneumoperitoneum was reconstructed, and a circular stapler (Ethicon Intraluminal Circular Staplers CDH29A; Ethicon, Guaynabo, Puerto Rico) was placed through the anus to complete the digestive tract reconstruction. Finally, the abdominal cavity was flushed and 2 plasma drainage tubes were placed through the perineum and the right lower abdomen (Fig. 5).

### 2.4. Statistical analysis

All statistical analyses were performed using the Statistical Package for the Social Sciences (SPSS®) version 23.0 (Chicago). Quantitative data were described by mean ± standard deviation if they obeyed normal distribution, otherwise they were described by median and upper and lower quartiles; qualitative data were described by number of cases and percentage. The comparison of quantitative data between 2 groups was based on whether the data obeyed normal distribution using Student *t*-test or Mann–Whitney *U* test, respectively. The comparison of qualitative data was based on whether the data were ordered using Mann–Whitney *U* test or Pearson chi-square test, and when the theoretical frequency was too small the Fisher exact probability method (Fisher exact test) instead of the chi-square test. *P* < .05 was considered statistically significant.

## 3. Results

### 3.1. Clinicopathological features

The conventional surgery group and the pushing the anus group consisted of 151 and 62 patients, respectively. Patients were 149 (70.0%) male and 64 (30.0%) female with a mean age of 62.5 ± 9.8 years. There was no significant differences between the 2 groups in regard to patients’age (*P* = .283), sex distribution (*P* = .267), body mass index (*P* = .504), the ASA classification (*P* = .560), the proportion of pathologic depth of invasion (*P* = .206), the lymph node metastasis (*P* = .927), the tumor size (*P* = .655), Neoadjuvant therapy (*P* = .508) and the distance from the lower edge of the tumor to the anal verge (*P* = .533) (Table 1).

**Table 1:** Clinicopathological features.

	Conventional surgery group (n = 151)	Pushing the anus group (n = 62)	*P*-value
Age, mean ± SD (yr)	62.0 ± 9.7	63.6 ± 10.1	.283
Sex			
Male	109	40	.267
Female	42	22	
BMI, mean ± SD (kg/m^2^)	19.1 ± 2.9	18.8 ± 3.0	.504
ASA classification			
I	78	29	.560
II	61	28	
III/IV	12	5	
PT stage			
T1	12	8	.206
T2	21	7	
T3	19	13	
T4	97	34	
PN stage			
N0	26	11	.927
N+	125	51	
Tumor size (cm)	3.6 (2.8–4.5)	3.5 (2.5–4.5)	.655
Neoadjuvant therapy	13	3	.508
Distance from the lower edge of the tumor to the anal verge	4.09 ± 0.61	4.03 ± 0.68	.533

ASA = American Society of Anesthesiology, SD = standard deviation.

### 3.2. Operative outcomes and postoperative recovery

**Figure 6.**
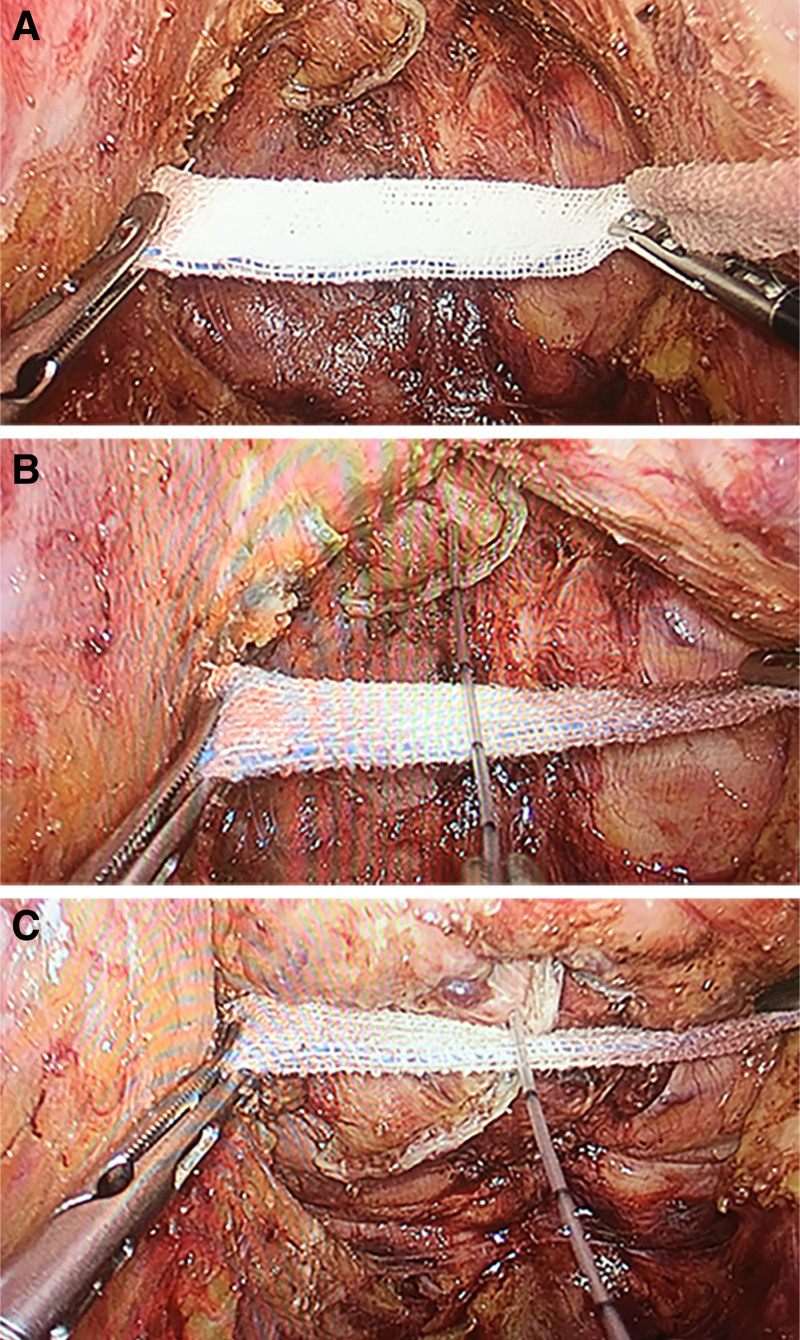
The distance of the distal rectum advance towards the pelvic inlet plane by pushing technique. (A) Placing the gauze strip in the pelvic inlet plane. (B) The distance from the distal rectum to the gauze strip before pushing the anus. (C) The distance from the distal rectum to the gauze strip after pushing the anus.

When pushing the anus forward from the perineum, we used an epidural anesthesia catheter (One scale represents 1 cm) to measure the distance of the distal rectum advanced to the pelvic inlet plane (Fig. 6A–C). In the pushing the anus group, the distal rectum was moved forward an average of 2.51 cm towards the pelvic inlet plane. Among them, male patients’ distal rectums were moved forward an average of 2.31 cm versus 2.89 cm in female patients. This may be due to the wider pelvis of female patients. There was no significant difference between the 2 groups in the aspect of the proportion of intraoperative bleeding (*P* = .701), the time of first postoperative meal (*P* = .218), the time of first anal exhaust (*P* = .139), the postoperative length of hospital stay (*P* = .340), the operative time (*P* = .884). However, compared with the conventional surgery group, the number of stapler cartridges was less in the pushing the anus group (*P* < .001) (Table 2).

**Table 2:** Operative outcomes and postoperative recovery compared between conventional surgery group and pushing the anus group.

	Conventional surgery group (n = 151)	Pushing the anus group (n = 62)	*P*-value
Intraoperative bleeding (mL)	50.0 (30.0–50.0)	50.0 (30.0–50.0)	.701
First postoperative meal (d)	5.0 (4.0–5.0)	5.0 (4.0–5.0)	.218
First anal exhaust (d)	3.0 (3.0–4.0)	3.0 (3.0–4.0)	.139
Postoperative hospital stay (d)	9.0 (9.0–10.0)	10.0 (9.0–12.0)	.340
Operative time (min)	249.3 ± 45.6	250.2 ± 39.7	.884
Number of stapler cartridges	2.0 (2.0–3.0)	1.0 (1.0–2.0)	<.001*
The average distance of anus moved forward (cm)			
All people		2.51	
Women		2.89	
Men		2.31	

* *P* < .05.

### 3.3. Postoperative complications

The statistical data showed that there was no significant difference in terms of the anastomotic bleeding (*P* = 1.000), the anastomotic stenosis (*P* = .582), the pulmonary complication (*P* = 1.000), the postoperative obstruction (*P* = 1.000), the urinary retention (*P* = 1.000), the Chylous ascites (*P* = .582) and the abdominal infection (*P* = .418). However, the incidence of anastomotic leakage in the pushing the anus group was much lower than that in the conventional surgery group (*P* = .043) (Table 3).

**Table 3:** Postoperative complication compared between conventional surgery group and pushing the anus group.

	Conventional surgery group (n = 151)	Pushing the anus group (n = 62)	*P*-value
Anastomotic leakage	17 (11.26%)	1 (0.02%)	.043*
Anastomotic bleeding	8 (5.30%)	3 (4.83%)	1.000
Anastomotic stenosis	2 (1.32%)	2 (3.22%)	.582
Pulmonary complication	11 (7.28%)	4 (6.45%)	1.000
Postoperative obstruction	5 (3.31%)	2 (3.22%)	1.000
Urinary retention	6 (3.97%)	2 (3.22%)	1.000
Chylous ascites	2 (1.32%)	2 (3.22%)	.582
Abdominal infection	4 (2.65%)	3 (4.83%)	.418

* *P* < .05.

## 4. Discussion

In this study, the anus was pushed forward from the perineum to make the surgical plane closer to the pelvic entrance to obtain wider surgical space, which reduced the number of stapler cartilages, as well as the incidence of anastomotic leakage. Due to the soft tissue structure of the pelvic floor and the anatomical position of the rectum, the distal rectum can be moved forward by pushing the anus of the patient who is in the Lloyd-Davies position. Although the distal rectum is moved forward only 2.51 cm on average, the increased operating space is huge because of the funnel-shaped anatomy of pelvis and the refinement and magnification characteristics of laparoscopic surgery. In addition, the lymph node metastasis rate of both groups in Table 1 is higher, while the rate of neoadjuvant therapy was lower. Some of the patients did have indications for neoadjuvant therapy, and detailed communication was conducted with the patients before surgery, but due to economic level, disease awareness and other factors, a considerable number of patients refused neoadjuvant therapy. Furthermore, the small sample size may also be 1 reason for this outcome.

Anal preservation of low-rectal cancer is still very difficult for men with narrow pelvis and obese patients. With the development of laparoscopy and robotic anastomosis technology, as well as the mature of ISR technology in recent years, it has become a reality to preserve the anus of rectal cancer patients whose tumor distance from the anal margin is <5 cm, especially for men with a narrow pelvis.^[14,15]^ In addition to the traditional laparotomy, the surgical methods mainly involve the robotic-assisted total mesorectal excision (RTME), the laparoscopic total mesorectal excision (LTME), and the transanal total mesorectal excision (taTME).^[16–18]^ It is not yet possible to prove which approach is more advantageous.^[19]^ Some studies suggest that robotic sphincter-saving total mesorectal excision can effectively improve the prognosis of rectal cancer patients.^[20]^ Some studies recommend taTME for obese male patients and low-rectal cancer.^[21,22]^ However, in some countries such as Norway, this procedure was discontinued due to a higher incidence of postoperative anastomotic leakage than nationwide, unfavorable local recurrence rates and growth patterns.^[23]^ Besides, taTME has a demanding learning curve, significant risk for morbidity and should be used only for selected patients.^[24]^ Some studies have demonstrated the superiority of robotic techniques over laparoscopy in mid- to low-rectal cancer patients, particularly in male patients, in terms of reduced bleeding, higher rates of mesorectal integrity, better long-term oncological outcomes, and improved functional outcomes.^[25]^ However, in the same conditions with female patients, no differences were found between LTME and RTME.^[26]^ At the same time, robotic surgery is currently difficult to implement because institutions need to be staffed with surgeons who have the appropriate skills and experience, so the LTME is still the main surgical method.^[19]^

In terms of the anastomotic leakage, how to reduce its incidence after low-rectal cancer surgery has always been a huge problem. Studies have reported that its incidence is 3% to 26%.^[27–29]^ In summary, there are several factors associated with it. Firstly, the distance between the tumor and the anal margin is an independent risk factor for it after laparoscopic sphincter-preserving surgery for rectal cancer.^[30,31]^ Secondly, some studies have shown that anastomotic leakage is related to the number of stapler cartilages. And 3 or more cartridges of the linear stapler are a risk factor for anastomotic leakage.^[32–34]^ Currently, the stapler cartridges of the linear staplers still cannot be rotated 90° because of its structure. In addition, due to the narrow space in the lower part of the funnel-shaped pelvis, the surgical dissection and the creation of an anastomosis is technically challenging. These all require additional stapler cartridges to complete the surgical procedure. What’ more, recent systematic evaluation has shown that using 2 cartridges also has a higher incidence of anastomotic leakage than using 1.^[35]^ In all our patients with anastomotic leaks, the number of stapler cartridges used was more than 2, which is consistent with the above research. Thirdly, the limited vascular supply is also an important risk factor of anastomotic leakage.^[36]^ It is reported that the more stapler cartridges used during the operation, the more the junction and length of the cutting edge, and the worse blood supply of the anastomosis.^[37]^

In this regard, during the rectal transection, we create an innovative method that pushing the anus forward from the perineum to bring the operating plane closer to the pelvic inlet. And a wider operating space can be obtained, so that the resection margin can be nearly perpendicular to the long axis of the rectum with the use of the linear staplers, which helps to reduce the number of stapler cartridges used in transverse rectal transection. In addition, the pushing technique can also reduce the tension during nailing to a certain extent, improve the nailing quality accordingly, and protect the integrity of the seromuscular layer and the blood supply of the broken end of the rectum better. All of these are helpful to reduce the occurrence of anastomotic leakage. Furthermore, this useful technique has the advantages to popularize due to its simplicity of operation.

However, as a single-center retrospective study, the sample size of this study is small, and its conclusions may be affected by factors such as race, regional economic level and living habits. Furthermore, the lower rate of anastomotic leakage in the group where the anus was pushed might be influenced by historical factors. In the future, multicenter retrospective studies with larger sample size and more reliable prospective study conclusions may bring new understanding of the application prospect of this technology.

## 5. Conclusion

In sphincter-preserving surgery for low-rectal cancer, pushing the anus forward from the perineum reduces the incidence of anastomotic leakage. This technique can be developed as a safer method for low-rectal cancer patients.

## Author contributions

**Conceptualization:** Qingqiang Yang.

**Data curation:** Rui Yang, Yuyi Yang.

**Formal analysis:** Rui Yang, Qingqiang Yang.

**Investigation:** Rui Yang.

**Visualization:** Yaxing Deng.

**Writing – original draft:** Rui Yang, Yuyi Yang, Yaxing Deng.

**Writing – review & editing:** Qingqiang Yang.

## References

[1] Sung H Ferlay J Siegel RL. Global cancer statistics 2020: GLOBOCAN estimates of incidence and mortality worldwide for 36 cancers in 185 countries. CA Cancer J Clin. 2021;71:209–49. 33538338 10.3322/caac.21660

[2] Rullier E Denost Q Vendrely V Rullier A Laurent C. Low rectal cancer: classification and standardization of surgery. Dis Colon Rectum. 2013;56:560–7. 23575394 10.1097/DCR.0b013e31827c4a8c

[3] Rullier E Laurent C Bretagnol F Rullier A Vendrely V Zerbib F. Sphincter-saving resection for all rectal carcinomas: the end of the 2-cm distal rule. Ann Surg. 2005;241:465–9. 15729069 10.1097/01.sla.0000154551.06768.e1 PMC1356985

[4] Tilney HS Tekkis PP. Extending the horizons of restorative rectal surgery: intersphincteric resection for low rectal cancer. Colorectal Dis. 2008;10:3–15; discussion 15. 17477848 10.1111/j.1463-1318.2007.01226.x

[5] Cahill RA Hompes R. Transanal total mesorectal excision. Br J Surg. 2015;102:1591–3. 26694990 10.1002/bjs.9933

[6] Quero G Fiorillo C Menghi R. Preliminary evaluation of two-row versus three-row circular staplers for colorectal anastomosis after rectal resection: a single-center retrospective analysis. Int J Colorectal Dis. 2022;37:2501–10. 36385574 10.1007/s00384-022-04283-8

[7] Zhao S Zhang L Gao F. Transanal drainage tube use for preventing anastomotic leakage after laparoscopic low anterior resection in patients with rectal cancer: a randomized clinical trial. JAMA Surg. 2021;156:1151–8. 34613330 10.1001/jamasurg.2021.4568 PMC8495603

[8] Rahbari NN Weitz J Hohenberger W. Definition and grading of anastomotic leakage following anterior resection of the rectum: a proposal by the International Study Group of Rectal Cancer. Surgery. 2010;147:339–51. 20004450 10.1016/j.surg.2009.10.012

[9] Lim SW Kim HR Kim YJ. Intracorporeal traction of the rectum with a beaded plastic urinary drainage bag hanger: comparison with conventional laparoscopic rectal cancer surgery. World J Surg. 2018;42:239–45. 28748421 10.1007/s00268-017-4153-x

[10] MacFarlane JK Ryall RD Heald RJ. Mesorectal excision for rectal cancer. Lancet. 1993;341:457–60. 8094488 10.1016/0140-6736(93)90207-w

[11] Schiessel R Karner-Hanusch J Herbst F Teleky B Wunderlich M. Intersphincteric resection for low rectal tumours. Br J Surg. 1994;81:1376–8. 7953423 10.1002/bjs.1800810944

[12] Aliyev V Goksoy B Goksel S. Intersphincteric resection for low rectal cancer: parameters affecting functional outcomes and survival rates. Surg Technol Int. 2021;39:166–72. 34699602

[13] Aliyev V Piozzi GN Bulut A. Robotic vs. laparoscopic intersphincteric resection for low rectal cancer: a case matched study reporting a median of 7-year long-term oncological and functional outcomes. Updates Surg. 2022;74:1851–60. 36198884 10.1007/s13304-022-01396-1

[14] Yang W Huang L Chen P. A controlled study on the efficacy and quality of life of laparoscopic intersphincteric resection (ISR) and extralevator abdominoperineal resection (ELAPE) in the treatment of extremely low rectal cancer. Medicine (Baltimore). 2020;99:e20245. 32481390 10.1097/MD.0000000000020245 PMC12245306

[15] Aliyev V Tokmak H Goksel S. Robotic Sphincter-saving total mesorectal excision for rectal cancer treatment: a single-surgeon experience in 103 consecutive male patients. Surg Technol Int. 2020;37:93–8. 32634247

[16] Aselmann H Kersebaum JN Bernsmeier A. Robotic-assisted total mesorectal excision (TME) for rectal cancer results in a significantly higher quality of TME specimen compared to the laparoscopic approach-report of a single-center experience. Int J Colorectal Dis. 2018;33:1575–81. 29971488 10.1007/s00384-018-3111-x

[17] Liu HC Li C Zhang F. Analysis on the technical characteristics and clinical efficacy of robotic-assisted intersphincteric resection for patients with low rectal cancer. Zhonghua Wei Chang Wai Ke Za Zhi. 2019;22:1137–43. 31874529 10.3760/cma.j.issn.1671-0274.2019.12.008

[18] Ren J Liu S Luo H Wang B Wu F. Comparison of short-term efficacy of transanal total mesorectal excision and laparoscopic total mesorectal excision in low rectal cancer. Asian J Surg. 2021;44:181–5. 32461015 10.1016/j.asjsur.2020.05.007

[19] Yamamoto S. Comparison of the perioperative outcomes of laparoscopic surgery, robotic surgery, open surgery, and transanal total mesorectal excision for rectal cancer: an overview of systematic reviews. Ann Gastroenterol Surg. 2020;4:628–34. 33319152 10.1002/ags3.12385 PMC7726682

[20] Aliyev V Tokmak H Goksel S. The long-term oncological outcomes of the 140 robotic sphincter-saving total mesorectal excision for rectal cancer: a single surgeon experience. J Robotic Surg. 2020;14:655–61. 10.1007/s11701-019-01037-7 31811567

[21] Vignali A Elmore U Milone M Rosati R. Transanal total mesorectal excision (TaTME): current status and future perspectives. Updates Surg. 2019;71:29–37. 30734896 10.1007/s13304-019-00630-7

[22] Adamina M Buchs NC Penna M Hompes R; St.Gallen Colorectal Consensus Expert Group. St.Gallen consensus on safe implementation of transanal total mesorectal excision. Surg Endosc. 2018;32:1091–103. 29234940 10.1007/s00464-017-5990-2 PMC5807525

[23] Wasmuth HH Faerden AE Myklebust T. Transanal total mesorectal excision for rectal cancer has been suspended in Norway. Br J Surg. 2020;107:121–30. 31802481 10.1002/bjs.11459

[24] Ourô S Ferreira M Roquete P Maio R. Transanal versus laparoscopic total mesorectal excision: a comparative study of long-term oncological outcomes. Tech Coloproctol. 2022;26:279–90. 35050434 10.1007/s10151-022-02570-8

[25] Aliyev V Goksel S Bakir B Guven K Asoglu O. Sphincter-saving robotic total mesorectal excision provides better mesorectal specimen and good oncological local control compared with laparoscopic total mesorectal excision in male patients with mid-low rectal cancer. Surg Technol Int. 2021;38:160–6. 33537982

[26] Aliyev V Piozzi GN Huseynov E. Robotic male and laparoscopic female sphincter-preserving total mesorectal excision of mid-low rectal cancer share similar specimen quality, complication rates and long-term oncological outcomes. J Robotic Surg. 2023;17:1637–44. 10.1007/s11701-023-01558-2 36943657

[27] Peeters KC Tollenaar RA Marijnen CA; Dutch Colorectal Cancer Group. Risk factors for anastomotic failure after total mesorectal excision of rectal cancer. Br J Surg. 2005;92:211–6. 15584062 10.1002/bjs.4806

[28] Jestin P Påhlman L Gunnarsson U. Risk factors for anastomotic leakage after rectal cancer surgery: a case-control study. Colorectal Dis. 2008;10:715–21. 18318752 10.1111/j.1463-1318.2007.01466.x

[29] Lee WS Yun SH Roh YN. Risk factors and clinical outcome for anastomotic leakage after total mesorectal excision for rectal cancer. World J Surg. 2008;32:1124–9. 18259805 10.1007/s00268-007-9451-2

[30] Gong JP Yang L Huang XE. Outcomes based on risk assessment of anastomotic leakage after rectal cancer surgery. Asian Pacific J Cancer Prev. 2014;15:707–12. 10.7314/apjcp.2014.15.2.707 24568483

[31] Frasson M Granero-Castro P Ramos Rodríguez JL; ANACO Study Group. Risk factors for anastomotic leak and postoperative morbidity and mortality after elective right colectomy for cancer: results from a prospective, multicentric study of 1102 patients. Int J Colorectal Dis. 2016;31:105–14. 26315015 10.1007/s00384-015-2376-6

[32] Ito M Sugito M Kobayashi A Nishizawa Y Tsunoda Y Saito N. Relationship between multiple numbers of stapler firings during rectal division and anastomotic leakage after laparoscopic rectal resection. Int J Colorectal Dis. 2008;23:703–7. 18379795 10.1007/s00384-008-0470-8

[33] Katsuno H Shiomi A Ito M. Comparison of symptomatic anastomotic leakage following laparoscopic and open low anterior resection for rectal cancer: a propensity score matching analysis of 1014 consecutive patients. Surg Endosc. 2016;30:2848–56. 26487228 10.1007/s00464-015-4566-2

[34] Sakamoto W Ohki S Kikuchi T. Higher modified Glasgow Prognostic Score and multiple stapler firings for rectal transection are risk factors for anastomotic leakage after low anterior resection in rectal cancer. Fukushima J Med Sci. 2020;66:10–6. 32074522 10.5387/fms.2019-17 PMC7269881

[35] Balciscueta Z Uribe N Caubet L. Impact of the number of stapler firings on anastomotic leakage in laparoscopic rectal surgery: a systematic review and meta-analysis. Tech Coloproctol. 2020;24:919–25. 32451807 10.1007/s10151-020-02240-7

[36] Gong W Li J. Combat with esophagojejunal anastomotic leakage after total gastrectomy for gastric cancer: a critical review of the literature. Int J Surg. 2017;47:18–24. 28935529 10.1016/j.ijsu.2017.09.019

[37] Braunschmid T Hartig N Baumann L Dauser B Herbst F. Influence of multiple stapler firings used for rectal division on colorectal anastomotic leak rate. Surg Endosc. 2017;31:5318–26. 28634627 10.1007/s00464-017-5611-0 PMC5715046

